# Estimating Local Diagnostic Reference Levels for Mammography in Dubai

**DOI:** 10.3390/diagnostics14010008

**Published:** 2023-12-20

**Authors:** Kaltham Abdulwahid Noor, Norhashimah Mohd Norsuddin, Muhammad Khalis Abdul Karim, Iza Nurzawani Che Isa, Wadha Alshamsi

**Affiliations:** 1 Dubai Health Academic Corporate, Radiology Department, Rashid Hospital, Dubai 00971, United Arab Emirates; p103342@siswa.ukm.edu.my; 2 Center of Diagnostic, Therapeutic and Investigative Studies (CODTIS), Faculty of Health Sciences, Universiti Kebangsaan Malaysia, Kuala Lumpur 56000, Malaysia; zawaniisa@ukm.edu.my; 3 Department of Physics, Faculty of Science, University Putra Malaysia, Serdang 43400, Malaysia; mkhalis@upm.edu.my; 4 SEHA, Medical Physics Department, Al Ain Hospital, Abu Dhabi 80050, United Arab Emirates; wshamsi@seha.ae

**Keywords:** diagnostic reference level, mean glandular dose, organ dose, mammography

## Abstract

As the total volume of mammograms in Dubai is increasing consistently, it is crucial to focus on the process of dose optimization by determining dose reference levels for such sensitive radiographic examinations as mammography. This work aimed to determine local diagnostic reference levels (DRLs) for mammography procedures in Dubai at different ranges of breast thickness. A total of 2599 anonymized mammograms were randomly retrieved from a central dose survey database. Mammographic cases for screening women aged from 40 to 69 years were included, while cases of breast implants and breast thickness outside the range of 20–100 mm were excluded. Mean, median, and 75 percentiles were obtained for the mean glandular dose (MGD) distribution of each mammography projection for all compressed breast thickness (CBT) ranges. The local DRLs for mammography in Dubai were found to be between 0.80 mGy and 0.82 mGy for the craniocaudal (CC) projection and between 0.89 mGy and 0.971.8 mGy for the mediolateral oblique (MLO) projection. Local DRLs were proposed according to different breast thicknesses, starting from 20 to 100 mm. All groups of CBT showed a slight difference in MGD values, with higher values in MLO views rather than CC views. The local DRLs in this study were lower than some other Middle Eastern countries and lower than the standard reference levels reported by the International Atomic Energy Agency (IAEA) at 3 mGy/view.

## 1. Introduction

Mammography is known for its advantages for early detection of breast cancer and its ability to lower the mortality rate by up to 30% [1]. Currently, mammography imaging is used for screening investigation of asymptomatic patients and diagnostic investigation for symptomatic patients. In the face of a growing demand for mammographic services, it is vital to ensure that this diagnostic modality is used safely and effectively. Concurrently, it is imperative to acknowledge that despite its benefits, the ionizing radiation from the modality exposure is associated with an increased risk of breast cancer among women, and, thus, the dose must be kept as low as reasonably achievable [1,2,3].

In this paper, we propose to design local DRLs for mammography in Dubai, a rapidly growing metropolitan area at the forefront of healthcare innovation that is striving to develop these levels specifically for mammography. 

The significance of establishing local DRLs for mammography in Dubai lies in the uniqueness of the population and the regional factors that may influence dose levels. Factors such as age, breast composition, and healthcare practices specific to Dubai can impact radiation doses and warrant a tailored approach to radiation protection. By considering these local factors, healthcare providers can ensure that radiation doses in mammography are appropriate, consistent, and in line with international best practices [2,4]. 

Several international organizations and regulatory bodies, such as the IAEA and national regulatory authorities, have issued guidelines and reference levels for mammography DRLs [5,6,7]. However, these international recommendations may not fully reflect the specific characteristics and needs of the population in Dubai. This is where the importance of local DRLs comes into play. By estimating and establishing specific DRLs tailored to the population and healthcare facilities in Dubai, medical practitioners can gain a more accurate understanding of the radiation doses delivered during mammography examinations. Hence, we aim in this study to provide valuable insights into the radiation doses delivered during mammography examinations in Dubai’s screening centers and compare them with international benchmarks [8]. 

The proposed DRLs serve as a guide for establishing national DRLs, ensuring that breast cancer screening programs are both effective and safe. By embracing this approach, the United Arab Emirates (UAE) can lead the way in providing high-quality breast healthcare services while adhering to international standards and best practices through moving forward toward the implementation of national DRLs. Currently, the lack of sufficient data on the MGD and organ calculation for mammography in Dubai is considered an alerting issue. This makes it difficult to establish local DRLs for mammography in Dubai, which is crucial to ensure that patients receive a safe dose of radiation during mammography procedures. Although there are some studies on establishing local DRLs for mammography in other countries around the United Arab Emirates, such as Australia, Malaysia, and Nigeria [9,10,11,12], these studies may not be applicable due to differences in patient populations, equipment, and protocols. Therefore, there is a need for a study to determine local DRLs for mammography in Dubai to ensure that patients receive the appropriate dose of radiation during mammography procedures. Also, this research study can be a guide to move forward toward estimating the national DRLs across the whole UAE. 

Generally, DRLs are derived by calculating the median value (75th or 95th percentile) of the distribution of MGD measurements from the observed sample [11]. The implementation of DRLs is both encouraged and essential for protection from medical radiation [13]. DRLs have been widely used in almost all radiography examinations all over the world. 

In August 2020, the last internal report on DRLs for mammography in Dubai considered one breast thickness. This study, however, will evaluate DRLs for different breast thicknesses as recommended by ICRP 135, and the MGD will be used as the dose quantity instead of the organ dose that was used in the preceding internal report issued by the Dubai Health Authority mammography facilities [14]. One of the primary reasons for relying on MGD instead of organ dose is that MGD provides a more accurate representation of the radiation dose received by the target tissue [2]. 

In mammography, the glandular tissue is the primary target for imaging and the tissue at risk for developing cancer. By focusing on the glandular dose, MGD accounts for the variation in breast composition, including the proportion of glandular and adipose tissue, which can differ among individuals. This consideration is important as breast density, which reflects the glandular tissue content, has been recognized as a significant risk factor for breast cancer. Therefore, by using MGD, the dose estimation is tailored to the specific tissue of interest and provides a more relevant indicator of potential cancer risk.

Additionally, the use of MGD allows for better comparison and benchmarking of dose levels across different facilities and regions. Organ dose, on the other hand, represents the dose to a specific organ, such as the breast, but does not differentiate between glandular and non-glandular components. Since the breast is composed of various tissues with different radio sensitivities, organ dose alone may not accurately reflect the radiation dose received by the glandular tissue. By using MGD, a standardized and consistent metric, it becomes possible to compare dose levels between facilities, track changes over time, and establish diagnostic reference levels specific to the local context. 

Apart from that, it is important to note that the assessment of breast dose plays a crucial role in ensuring the quality of mammography using X-ray technology. The United Kingdom (UK) established a standard protocol for dosimetry in conventional mammography using 2D projection in 1989. In 2005, this protocol was expanded and reported by the Institute of Physics and Engineering in Medicine (IPEM) to account for the use of X-ray spectra from different target/filter combinations through the introduction of an “s-factor”. Additionally, a “c-factor” was introduced to provide dosimetry for a range of breast granularities [15]. 

Similar methodologies have been adopted in the European Conference on Technology Enhanced Learning held in 1996 and 2006 and the International Atomic Energy Agency (IAEA) protocols [15,16,17]. All three protocols utilize conversion factors to relate measurements of the incident air kerma at the upper surface of the breast to the mean dose to the glandular tissue within the breast (known as MGD), which is derived from Monte Carlo calculations developed by Dance in 1990 [15]. Hence, the present study seeks to establish the local DRLs for screening mammography in Dubai, considering MGD as the dose quantity based on different thicknesses of the breast.

## 2. Materials and Methods

**Figure 1:**
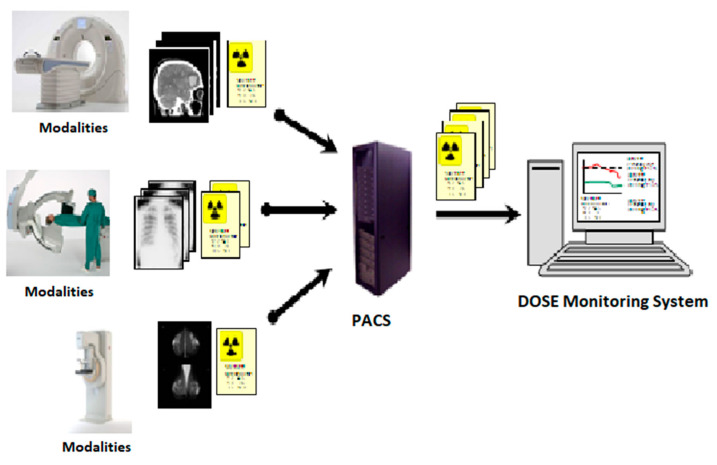
The flow chart depicting the Dose TQM mechanism system.

The ethical approval for this cross-sectional study was granted by both the Dubai Scientific Research Ethics Committee (DSREC-SR-08/2022_04) as well as the Medical Research and Ethics Committee at the National University of Malaysia UKM (JEP-2022-622). The dose survey data of patients who underwent mammographic procedures between November 2019 and November 2022 was retrospectively collected using the DOSE TQM system (Qaelum NV, Leuven, Belgium) and extracted into an SPSS software version 25.0 for inferential analysis. This system is directly integrated with the picture archiving and communication system (PACS) used in Dubai’s radiology departments. The system framework and its connection to PACS are illustrated in Figure 1.

A total of 2599 mammograms was extracted from the DOSE TQM system. Patients’ data, such as age, breast thickness, and projections, and exposure parameters, such as kilovolt peak (kVp), milliampere-seconds (mAs) target, filter, angulation degree, and half value level (HVL), were extracted for dose calculations.

To capture a diverse sample and provide valuable insights into mammography practices in Dubai, women aged 40–69 years were included and aligned with the target population for five breast cancer screening radiology departments in Dubai. Women with breast implants were excluded from the analysis due to the potential impact of implants on mammographic images and the estimation of dose. In this study, only standard mammography projections like the MLO and CC for both breasts were included. Supplementary projections, such as laterals, mediolateral (ML), or lateromedial (LM) projections and lateromedial oblique (LMO) were excluded. This is because, in the present study, the aim was to ascertain the DRLs of mammography by examining data from two standard projections which are commonly used, especially in the screening protocols. 

As discussed earlier, this study focuses on estimating local DRLs for mammography in the city of Dubai. Local DRLs, in particular, pertain to radiation dose levels that are tailored to the demographic, clinical, and technological attributes of a specific region or healthcare facility. They are derived from widespread data collected from the locality, considering factors such as patient populations, equipment variations, and clinical practices unique to the area. In our case, data were extracted from five screening centers in Dubai, offering a clear understanding of the radiation exposure levels dominant within this specific healthcare environment.

On the other hand, national DRLs provide a more generalized standard established at the country-wide level. They serve as primary benchmarks and are typically based on broad-scale surveys encompassing a wide array of healthcare institutions across the nation. While national DRLs offer a valuable baseline for comparison, they may not always align perfectly with the complexities of a local healthcare system. Therefore, relying solely on national DRLs may not capture the characteristics of the radiological practices and patient demographics specific to Dubai.

MGD or average glandular dose (AGD) is a measure utilized to quantify the amount of radiation absorbed by the breast during mammography. This value is estimated using standard breast parameters, such as the entrance surface air kerma (ESAK) and HVL [18]. The MGD was determined individually using the approach developed by Dance [15,19]. The methodology employed by Dance is elucidated in the following formula.

MGD = Kgcs

where:

○ K is the mammography machine output (calibration) measured in mGy. It is also known as the entrance dose at the surface of the breast. This quantity was provided by the manufacturers for each mammography scan and could also be obtained from the DICOM header;
○ g is a conversion factor describing the fraction of “K” that is absorbed by the glandular tissue in the breast. g depends on breast thickness and the HVL;
○ c is a correction factor for breast composition that corrects for any difference in granularity from 50%, i.e., from 0 to 100%. Dance et al. [15,19] provided a reference table of c factors for various HVLs, breast thicknesses from 2 to 11 cm, and granularity from which one can extrapolate the percentage of granularity for everyone;
○ s is a correction factor for the X-ray spectrum that can be altered when using different target and filter combinations. Such a correction factor is independent of the HVL and can be found in a simple reference table that includes various target and filter combinations [10].

Air kerma was calculated based on the actual exposure parameters, along with breast thickness data that had been adjusted according to tube output measurements. Conversion factors were derived using the information on beam quality, anode/filter/HVL, and corrected breast thickness [20]. Since conversion factors are only available for a specific thickness and HVL, linear interpolation was utilized. Data were categorized into two mammographic projection groups: (1) MLO and (2) CC. CBT, on the other hand, was categorized into four groups: (1) 20 to 39 mm, (2) 40 to 59 mm, (3) 60 to 70 mm, and (4) 80 to 100 mm.

**Figure 2:**
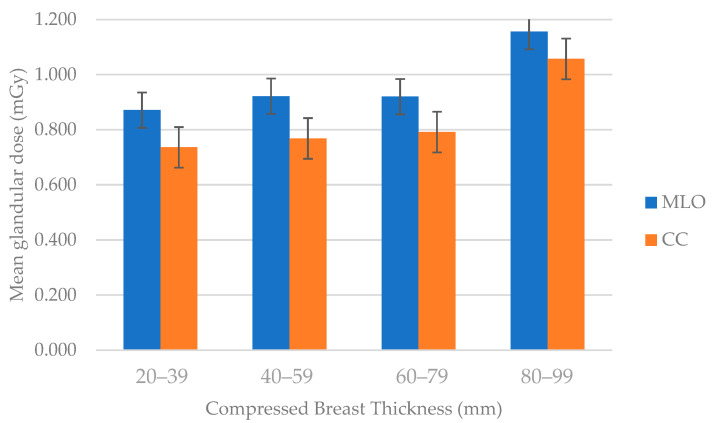
Distribution of MGD (mGy) to breast thickness at different mammographic projections. MGD increased with higher breast thickness for both projections in MLO and CC.

For DRL calculation, the mean MGD/view of CC and MLO were summed up and divided by two to obtain the median MGD. Next, the mean, median, and 75th percentile of MGD for each mammographic projection were computed [21] (see Figure 2). The 75th percentile values were obtained for the MGD of both mammography projections to estimate the local DRLs and tabulated separately against four CBT groups for five health institution centers. 

All data were then analyzed using SPSS version 25. The data were tested for normality using objective and subjective means, subjectively by visual observation using histograms and objectively using the Kolmogorov–Smirnov (KS) test before inferential statistics. Descriptive statistics are used to acquire mean, median, standard deviation, percentiles, and range. The differences between MGDs across different CBT groups were tested using the Kruskal–Wallis test. The Mann–Whitney U test was used to compare MDG and DRLs between MLO and CC, as the normality test for this data shows no normal distribution. A *p* value less than 0.05 was considered statistically different. 

## 3. Results

### 3.1. Target and Filter and HVL across All Health Centers

Table 1 shows that all the health centers and the hospital utilize tungsten or molybdenum as the target/filter material for mammography. The HVL ranges from 0.09 to 0.43, indicating the thickness of the material required to attenuate the tungsten radiation by half. The HVL mean values range from 0.15 to 0.18, with standard deviations of 0.04 to 0.05, reflecting the consistency of the measurements within each facility.

### 3.2. Scanning Parameters across Different CBTs for MLO and CC Projections

From the data, we can observe that as CBT increases, the compression force, tube voltage, tube current, and entrance dose also tend to increase for both MLO and CC projections. The results for MLO revealed distinct trends with increasing CBT. For the CBT range of 20–39 mm (*N* = 83), the mean compression force applied was 105.3 ± 42.3 N, the mean tube voltage used was 26.9 ± 0.7 kV, the mean tube current was 63.1 ± 21.8 mAs, and the mean entrance dose was 1.0 ± 0.3 mGy. For the breast thickness range of 40–59 mm (*N* = 936), the mean compression force applied was 110.9 ± 42.4 N, the mean tube voltage used was 28.8 ± 0.6 kV, the mean tube current was 96.1 ± 33.9 mAs, and the mean entrance dose was 1.3 ± 0.4 mGy. For the CBT range of 60–79 mm (*N* = 1409), the mean compression force applied was 116.7 ± 42.6 N, the mean tube voltage used was 30.4 ± 0.5 kV, the mean tube current was 129.2 ± 41.5 mAs, and the mean entrance dose was 1.4 ± 0.4 mGy. For the CBT range of 80–99 mm (*N* = 171), the mean compression force applied was 119.4 ± 46.2 N, the mean tube voltage used was 31.9 ± 0.5 kV, the mean tube current was 178.2 ± 61.0 mAs, and the mean entrance dose was 1.9 ± 0.6 mGy. As CBT increased, the compression force applied during mammography showed a significant increment (*p* < 0.001), suggesting that greater force is required to achieve optimal image quality in thicker breasts. Similarly, we observed statistically significant increments in tube voltage (*p* < 0.001 *) and tube current (*p* < 0.001) as CBT increased, indicating the need for adjustments in imaging settings to accommodate varying breast thicknesses. Additionally, the entrance dose, a crucial consideration for patient safety, demonstrated a significant rise (*p* < 0.001) with increasing breast thickness (Table 2).

For the CC at the CBT range of 20–39 mm (*N* = 115), the mean CBT was 33.9 ± 5.5 mm, the mean age was 51.8 ± 7.7 years, the mean compression force applied was 118.9 ± 43.0 N, the mean tube voltage used was 26.9 ± 0.6 kV, the mean tube current was 64.8 ± 21.9 mAs, and the mean entrance dose was 0.9 ± 0.3 mGy. For the CBT range of 40–59 mm (*N* = 1356), the mean breast thickness in the CC view was 51.5 ± 5.3 mm, with a mean age of 51.6 ± 7.1 years. The mean compression force applied was 115.8 ± 43.1 N, the mean tube voltage used was 28.8 ± 0.6 kV, the mean tube current was 89.7 ± 29.2 mAs, and the mean entrance dose was 1.2 ± 0.4 mGy. For the CBT range of 60–79 mm (*N* = 1076), the mean CBT in the CC view was 66.3 ± 4.9 mm, with a mean age of 51.2 ± 6.6 years. The mean compression force applied was 112.4 ± 42.4 N, the mean tube voltage used was 30.3 ± 0.5 kV, the mean tube current was 115.9 ± 62.9 mAs, and the mean entrance dose was 1.3 ± 0.4 mGy. For the CBT range of 80–99 mm (*N* = 52), the mean CBT in the CC view was 83.4 ± 3.7 mm, with a mean age of 50.9 ± 6.3 years. The mean compression force applied was 107.4 ± 44.3 N, the mean tube voltage used was 31.8 ± 0.6 kV, the mean tube current was 159.4 ± 62.9 mAs, and the mean entrance dose was 1.7 ± 0.6 mGy. 

The results of the one-way ANOVA revealed significant differences among the imaging parameters across all CBTs, while compression force did not show statistically significant variations (*p* = 0.092) across the CBT ranges. There were significant differences in tube voltage (*p* < 0.001), tube current (*p* < 0.001), and entrance dose (*p* < 0.001). These findings indicate that breast thickness has a notable impact on tube voltage, tube current, and entrance dose but not on compression force (Table 2).

### 3.3. MGD across Breasts Thickness Range

Figure 2 demonstrates a clear trend in both MLO and CC views, where the MGD generally increases with an increase in CBT. In the MLO view, the MGD values were 0.87, 0.92, 0.92, and 1.16 for breast thickness ranges of 20–39 mm, 40–59 mm, 60–79 mm, and 80–99 mm, respectively. Similarly, in the CC view, the MGD values were 0.74, 0.77, 0.79, and 1.06 for the same CBT groups.

### 3.4. DRLs for MLO and CC Projections across Different CBTs 

The results of the Kruskal–Wallis test, examining the impact of breast thickness on various imaging parameters in MLO projection of mammography, are presented in Table 3. For the CBT range of 20–39 mm (*N* = 83), the measured parameter ranged from 0.4 to 2.8, with a mean of 0.87 (±0.34) and a median of 0.79 (interquartile range—IQR: 0.37). Similarly, for the CBT range of 40–59 mm (*N* = 936), the measured parameter ranged from 0.4 to 2.3, with a mean of 0.92 (±0.32) and a median of 0.85 (IQR: 0.41). The same trend was observed for the CBT ranges of 60–79 mm (*N* = 1409) and 80–99 mm (*N* = 171), with mean values of 0.92 (±0.29) and 1.16 (±0.48), and median values of 0.85 (IQR: 0.33) and 1.04 (IQR: 0.40), respectively. The Kruskal–Wallis test showed a statistically significant difference between the groups (*p* < 0.001). Notably, at the 75th percentile, the measured parameter values are 0.90, 0.95, 0.95, and 0.97 for the respective CBT ranges of 20–39 mm, 40–59 mm, 60–79 mm, and 80–99 mm. These values signify the data point below which 75% of the data falls. Strikingly, the 75th percentile values, which are considered to be local diagnostic reference levels, exhibit an increasing trend with higher CBT ranges, indicating a potential correlation between the measured parameter and breast thickness (Table 3).

For the CBT range of 20–40 mm (*N* = 115), the MGD ranged from 0.3 to 2.0, with a mean of 0.74 (±0.27) and a median of 0.68 (interquartile range—IQR: 0.32). For the CBT range of 40–60 mm (*N* = 1356), the MGD ranged from 0.0 to 2.6, with a mean of 0.77 (±0.30) and a median of 0.69 (IQR: 0.35). For the CBT range of 60–80 mm (*N* = 1076), the MGD ranged from 0.4 to 1.9, with a mean of 0.79 (±0.26) and a median of 0.73 (IQR: 0.31). For the CBT range of 80–99 mm (*N* = 52), the MGD ranged from 0.7 to 2.5, with a mean of 1.06 (±0.39) and a median of 0.94 (IQR: 0.37). A noticeable trend can be observed in the MGD values with respect to CBT ranges. As the breast thickness increases, there appears to be a trend of higher MGD values. The CBT range of 20–40 mm has the lowest mean MGD value of 0.74, while the CBT range of 80–99 mm has the highest mean MGD value of 1.06. Similarly, the medians follow a similar pattern, with the lowest value in the 20–40 mm range (0.68) and the highest value in the 80–99 mm range (0.94). The Kruskal–Wallis test showed a statistically significant difference between the groups (*p* < 0.001 *). 

For the MLO, we observed an increasing trend in MGD as CBT increased. The 75th percentile MGD values ranged from 0.895 to 0.967 for the CBT ranges of 20–40 mm to 80–99 mm, respectively. Similarly, in the CC, we identified a trend of increasing MGD values with increasing CBT. 

The 75th percentile MGD values ranged from 0.815 to 0.825 mGy for the CBT ranges of 20–40 mm to 80–99 mm, respectively. Regarding mammographic projections, DRLs for MLO ranged from 0.895 to 0.967 mGy for CBT of 20–30 mm to 80–99 mm, respectively. Whilst for CC, DRLs ranged from 0.815 to 0.825 mGy, lower than MLO for all CBT groups. This indicates that higher radiation doses may be delivered to the glandular tissues of the breast in the CC view for thicker breasts. These findings indicate a correlation between breast thickness and MGD, with thicker breasts generally associated with higher radiation doses (Table 3).

### 3.5. Mean Glandular Dose for MLO and CC Projections at Different Healthcare Centers

For all centers combined, the MGD range in the MLO was 0.4–5.1, with a mean of 0.93 (±0.33) and a median of 0.86 (IQR: 0.36). While for CC, the MGD range was 0.0–2.6, with a mean of 0.78 (±0.29) and a median of 0.72 (IQR: 0.34). When considering all centers combined, a consistent pattern emerges. In both MLO and CC views, as CBT increases, the MGD values also show a steady rise. When comparing the data across health centers, Center 2 has the highest MGD for MLO projection (1.06 ± 0.33 mGy), followed by Center 5 (1.01 ± 0.37 mGy), Center 1 (0.87 ± 0.28 mGy), Center 4 (0.84 ± 0.29 mGy), and Center 3 (0.77 ± 0.23 mGy), whilst for CC, Center 5 (0.96 ± 0.39 mGy) had the highest MGD, followed by Center 2 (0.84 ± 0.27 mGy), Center 1 (0.75 ± 0.24 mGy), Center 4 (0.68 ± 0.22 mGy), and Center 3 (0.65 ± 0.19 mGy). 

Table 4 exhibits MGD for all health centers in Dubai at MLO and CC projections. At Center 1, the MGD range in the MLO was 0.5–2.5 mGy, with a mean of 0.87 ± 0.28 mGy and a median of 0.79 mGy (IQR: 0.29). For CC, the MGD range was 0.4–2.3 mGy, with a mean of 0.75 ± 0.24 mGy and a median of 0.69 mGy (IQR: 0.29). At Center 2, the MGD range in the MLO was 0.4–3.1 mGy, with a mean of 1.06 ± 0.33 mGy and a median of 0.96 mGy (IQR: 0.37). For CC, the MGD range was 0.3–1.9 mGy, with a mean of 0.84 ± 0.27 mGy and a median of 0.78 mGy (IQR: 0.32). At Center 3, the MGD range in the MLO was 0.5–1.8 mGy, with a mean of 0.77 mGy (±0.23) and a median of 0.69 mGy (IQR: 0.19). For CC, the MGD range was 0.4–1.3 mGy, with a mean of 0.65 ± 0.19 mGy and a median of 0.62 mGy (IQR: 0.24). At Center 4, the MGD range in the MLO was 0.4–5.1 mGy, with a mean of 0.84 ± 0.29 mGy and a median of 0.78 mGy (IQR: 0.31). For CC, the MGD range was 0.3–1.8 mGy, with a mean of 0.68 ± 0.22 mGy and a median of 0.63 mGy (IQR: 0.29). At Center 5, the MGD range in the MLO was 0.5–2.8 mGy, with a mean of 1.01 ± 0.37 mGy and a median of 0.95 mGy (IQR: 0.46). For CC, the MGD range was 0.0–2.6 mGy, with a mean of 0.96 ± 0.39 mGy and a median of 0.89 mGy (IQR: 0.52). When examining the data for individual healthcare centers, similar trends persist. In most cases, MGD values increase with increasing breast thickness both in the MLO and CC views. This trend is consistent across different centers and further supports the notion that breast thickness plays a significant role in determining the radiation doses delivered during mammography.

## 4. Discussion 

The data analysis from this study considering all five screening centers combined, illustrated that the 75th percentile MGD values are consistently higher in the MLO (0.95 mGy) compared to the CC (0.78 mGy), as in the table below.

While opting for a specific breast thickness to represent an entire population may seem like a simple approach, it may not be entirely accurate due to the heterogeneity of the population and the possible range of breast thicknesses, which can vary from 1 to 10 cm [22]. To enhance the effectiveness of mammography’s DRL procedure, determining DRL values for different breast thicknesses is a highly complex yet more reliable technique. Breast thickness varies across different regions of the world, with women in the Asia-Pacific region having thinner and denser breasts compared to those in Europe and North America [16,23]. Conversely, women in North America generally have thicker and denser breasts than women in Europe. This variation in breast thickness is a result of geographical differences and highlights the need for stratified DRLs based on different compressed breast thickness ranges [17]. The ICRP recommends setting DRLs for a single standard breast thickness or a range of breast thicknesses [14,24]. 

Table 5 proves the above information, which is the correlation between breast thickness and local DRLs. It gives a detailed estimation of the local DRLs according to different breast thicknesses in two main projections, as described in the table below.

Although recommended by the ICRP, this approach has been adopted with slight variations in many countries. The findings indicate that the proposed DRLs are slightly lower than those in Australia and France [25]. The comparative analysis of DRLs in mammography between Dubai and other countries reveals distinct variations in radiation dose practices and protocols. Studies demonstrate that DRLs in Dubai may differ from international benchmarks due to factors such as patient demographics, screening and diagnostic practices, and equipment variations. Variability in mammography imaging parameters, such as compression force and exposure settings, can contribute to differences in DRLs. Additionally, variations in national guidelines and regulatory frameworks impact the establishment and implementation of DRLs in mammography. Understanding these differences can help in developing tailored approaches for dose optimization in mammography, ensuring optimal image quality while minimizing patient radiation exposure and promoting breast health.

While breast thickness is an essential factor to consider, it is crucial to acknowledge that there are other factors that can significantly influence mammographic outcomes. These factors include age, breast composition, and specific healthcare practices, which should not be overlooked or assumed to have minimal impact.

Age is an important variable to consider in mammography as breast tissue changes with age. Younger women typically have denser breasts, while older women tend to have more fatty tissue. The differences in breast composition can affect the radiation dose distribution and image quality. Therefore, it is important to account for age as a potential confounding factor in the statistical analysis. Stratifying the data based on age groups or including age as a co-variate in the analysis can help control for its influence on mammographic outcomes.

Breast composition is another critical factor that can vary among individuals. It includes the proportion of glandular and adipose tissue in the breast, which can affect the image quality and the radiation dose delivered. Women with higher breast density have a higher proportion of glandular tissue, making it more challenging to detect abnormalities and potentially requiring higher radiation doses for optimal visualization. Considering breast composition as a variable in the statistical analysis allows for a more comprehensive understanding of its impact on mammographic outcomes.

In addition to age and breast composition, specific healthcare practices can also influence mammographic outcomes. These practices may include positioning techniques, compression protocols, and equipment calibration. Variations in these practices can lead to differences in radiation dose, image quality, and ultimately the diagnostic accuracy of mammograms. It is crucial to control for and consider these healthcare practices as potential confounding factors in the statistical analysis to ensure that the results accurately reflect the impact of breast thickness on mammographic outcomes. 

Apart from factors affecting the diagnostic reference levels in mammography, there is limited information on the current national DRLs for mammography in Arab countries. However, some studies have been conducted in Saudi Arabia to estimate cancer risks during mammography procedures and establish local DRLs for mammography. In 2018, a study was conducted in Saudi Arabia to establish a DRL for mammography to minimize the malignancy risk due to ionizing radiation. The study proposed a DRL of 2.5 mGy for breast thicknesses between 4 and 8 cm [26]. In the UAE, a nationwide dose survey was conducted in 2017 to obtain DRL data for dental radiography, mammography, computed tomography (CT), and nuclear medicine. However, the results of this survey have not been published yet. A study conducted in 2015 investigated DRLs for mammography in different countries in the Latin American region, including Brazil, Chile, Costa Rica, El Salvador, Mexico, Paraguay, and Venezuela. The study found that the DRLs for mammography varied widely among countries, ranging from 1.5 to 3.5 mGy [17,27]. For example, in Malaysia, the local DRLs for full-field digital mammography (FFDM) and digital breast tomosynthesis (DBT) were established at different CBT ranges, with DBT having significantly higher AGD than FFDM [11]. In Thailand, the local DRLs for FFDM and DBT were found to be 1.65 mGy and 1.89 mGy, respectively, and were lower than the standard reference levels reported by the IAEA [28]. In Morocco, the local DRLs for FFDM and DBT were 1.7 mGy and 1.8 mGy, respectively, and were compared with local DRLs from other countries [29]. In Ghana, the local DRLs for digital mammography were higher than international studies, indicating a need for dose optimization [30].

To ensure dose optimization, it is always encouraged that each country start calculating its own local DRLs for all radiographic procedures and then move forward toward designing national guidelines and benchmarking with international standards. In addition to this, it is important to note that DRLs should be reviewed periodically and updated to reflect advancements in technology, changes in clinical practices, and evolving dose-reduction techniques. Continuous monitoring and benchmarking of dose levels are crucial to ensure the ongoing optimization of radiation protection for patients undergoing diagnostic imaging procedures [31].

## 5. Conclusions

By focusing on local DRLs, this study sheds light on the unique radiological landscape of Dubai, offering a more refined and contextually relevant assessment of mammographic radiation doses in this setting. For the first time, this research proposed local DRLs for mammography according to different breast thicknesses, as recommended by ICRP. Healthcare providers in Dubai can ensure that diagnostic images are of the highest quality by tailoring DRLs to specific regional characteristics and practices in addition to optimizing radiation doses. This localization would lead to more accurate and personalized dose-optimization strategies, improving the accuracy and reliability of mammographic examinations while minimizing unnecessary radiation exposure. By applying the proposed local DRLs, it would enhance patient safety, contribute to better diagnostic accuracy, and support advancements in breast health within the unique healthcare environment of Dubai. Moreover, safety authorities within the country may use the findings of this study as an initial guide to design the national guidelines for the country, which will probably improve the breast screening imaging protocols in terms of dose optimization as well as image quality.

## Figures and Tables

**Table 1:** The target and filter in Mammographic studies in five Dubai health centers.

Health Centers	*N*	Target/Filter	HVL Range	HVL Mean ± SD
Center 1	701	Tungsten	0.09–0.43	0.17 ± 0.04
Center 2	711	Tungsten	0.09–0.43	0.18 ± 0.05
Center 3	50	Tungsten	0.13–0.31	0.18 ± 0.04
Center 4	748	Tungsten	0.10–0.43	0.15 ± 0.04
Center 5	389	Tungsten/Molybdenum	0.09–0.43	0.18 ± 0.05

HVL, half value level; SD, standard deviation.

**Table 2:** Patient characteristics and scanning parameters across breast thicknesses for MLO and CC projections (*n* = 2599).

Mammographic Projections	Compressed Breast Thickness (mm)	*N*	Thickness (mm)	Age (y/o)	Compression Force (N)	Tube Voltage (kV)	Tube Current (mAs)	Entrance Dose (mGy)
MLO	20–39	83	33.5 ± 5.6	50.4 ± 7.6 (41–67)	105.3 ± 42.3 (40.7–190.7)	26.9 ± 0.7 (24–28)	63.1 ± 21.8 (33–148)	1.0 ± 0.3 (0.6–2.3)
	40–59	936	52.2 ± 5.2	51.5 ± 7.2 (41–68)	110.9 ± 42.4 (31.2–198.1)	28.8 ± 0.6 (28–30)	96.1 ± 33.9 (35–267)	1.3 ± 0.4 (0.6–3.1)
	60–79	1409	67.8 ± 5.3	51.5 ± 6.8 (41–68)	116.7 ± 42.6 (28.0–193.8)	30.4 ± 0.5 (30–32)	129.2 ± 41.5 (60–430)	1.4 ± 0.4 (0.7–4.4)
	80–99	171	84.8 ± 4.5	50.9 ± 6.3 (41–68)	119.4 ± 46.2 (33.4–189.2)	31.9 ± 0.5 (24–32)	178.2 ± 61.0 (94–406)	1.9 ± 0.6 (1.04–4.04)
One-way ANOVA				0.395	0.001 *	<0.001 *	<0.001 *	<0.001 *
CC	20–39	115	33.9 ± 5.5	51.8 ± 7.7 (41–68)	118.9 ± 43.0 (40.7–191.7)	26.9 ± 0.6 (24–28)	64.8 ± 21.9 (31–124)	0.9 ± 0.3 (0.6–1.7)
	40–59	1356	51.5 ± 5.3	51.6 ± 7.1 (41–68)	115.8 ± 43.1 (31.2–198.1)	28.8 ± 0.6 (28–30)	89.7 ± 29.2 (35–237)	1.2 ± 0.4 (0.6–3.5)
	60–79	1076	66.3 ± 4.9	51.2 ± 6.6 (41–68)	112.4 ± 42.4 (28.0–191.4)	30.3 ± 0.5 (30–32)	115.9 ± 62.9 (57–269)	1.3 ± 0.4 (0.7–3.0)
	80–99	52	83.4 ± 3.7	50.9 ± 6.3 (41–68)	107.4 ± 44.3 (33.8–183.2)	31.8 ± 0.6 (30–32)	159.4 ± 62.9 (96–424)	1.7 ± 0.6 (1.1–3.6)
One-way ANOVA				0.413	0.092	<0.001	<0.001	<0.001

CC, craniocaudal; MLO, mediolateral Oblique; ANOVA, analysis of variance; * statistically significant differences at *p* value < 0.05.

**Table 3:** The range, mean, median, 25th percentile, 50th percentile, and 75th percentile of MGD for MLO and CC projections in different CBT groups.

Mammographic Projection	Compress Breast Thickness (mm)	*N*	Mean ± SD	Median (IQR)	Kruskal–Wallis Test	25th Percentile	50th Percentile	75th Percentile
MLO	20–39	83	0.87 ± 0.34	0.79 (0.37)	<0.001 *	0.612	0.786	0.895
	40–59	936	0.92 ± 0.32	0.85 (0.41)		0.615	0.794	0.951
	60–79	1409	0.92 ± 0.29	0.85 (0.33)		0.643	0.779	0.949
	80–99	171	1.16 ± 0.48	1.04 (0.40)		0.629	0.804	0.967
CC	20–40	115	0.74 ± 0.27	0.68 (0.32)	<0.001 *	0.496	0.638	0.815
	40–60	1356	0.77 ± 0.30	0.69 (0.35)		0.503	0.643	0.805
	60–80	1076	0.79 ± 0.26	0.73 (0.31)		0.526	0.645	0.802
	80–99	52	1.06 ± 0.39	0.94 (0.37)		-	0.708	0.825

CC, craniocaudal; MLO, Mediolateral Oblique; IQR, interquartile range; * statistically significant differences at *p* value < 0.05.

**Table 4:** The range, mean, median, 25th percentile, 50th percentile and 75th percentile of MGD for MLO and CC projections at different centers.

Center	Mammographic Projections	Mean ± SD	Median (IQR)	Mann–Whitney *U* Test	25th Percentile	50th Percentile	75th Percentile
All	MLO	0.93 ± 0.33	0.86 (0.36)	<0.001 *	0.627	0.784	0.951
	CC	0.78 ± 0.29	0.72 (0.34)		0.557	0.652	0.785
Center 1	MLO	0.87 ± 0.28	0.79 (0.29)	<0.001 *	0.626	0.772	0.946
	CC	0.75 ± 0.24	0.69 (0.29)		0.575	0.655	0.813
Center 2	MLO	1.06 ± 0.33	0.96 (0.37)	<0.001 *	0.651	0.811	0.948
	CC	0.84 ± 0.27	0.78 (0.32)		0.574	0.679	0.764
Center 3	MLO	0.77 ± 0.23	0.69 (0.19)	<0.001 *	0.640	0.772	1.031
	CC	0.65 ± 0.19	0.62 (0.24)		0.530	0.638	0.891
Center 4	MLO	0.84 ± 0.29	0.78 (0.31)	<0.001 *	0.623	0.777	0.951
	CC	0.68 ± 0.22	0.63 (0.29)		0.520	0.618	0.732
Center 5	MLO	1.01 ± 0.37	0.95 (0.46)	<0.001 *	0.623	0.791	0.964
	CC	0.96 ± 0.39	0.89 (0.52)		0.631	0.702	0.934

CC, craniocaudal; MLO, Mediolateral Oblique; IQR, interquartile range; SD, standard deviation; * statistically significant differences at *p* value < 0.05.

**Table 5:** Estimated local DRLs according to breast thickness in two main mammographic projections.

Mammographic Projection	CBT Ranges (mm)	Estimated Local DRLs (mGy)
MLO	20–39	0.89
	40–59	0.95
	60–79	0.95
	80–99	0.97
CC	20–30	0.81
	40–56	0.80
	60–79	0.80
	80–99	0.82

MLO, mediolateral oblique; CC, craniocaudal; CBT, compressed breast thickness; DRLs, diagnostic reference levels.

## Data Availability

The data presented in this study are available on request from the corresponding author. The data are not publicly available for ethical purposes.
